# Comparison of Online Peer‐Assisted Learning and Faculty‐Led Teaching for Short Answer Questions

**DOI:** 10.1111/tct.70069

**Published:** 2025-03-18

**Authors:** Gurtek Singh Samra, Kashmir Gaddu, Joseph Ryan Wong Sik Hee, Krupali Brahmbhatt, David Bowrey, Max Seabrook

**Affiliations:** ^1^ Department of Medicine University of Leicester Leicester UK; ^2^ Department of Clinical Education University Hospitals of Leicester Leicester UK; ^3^ Department of Surgery University Hospitals of Leicester Leicester UK

**Keywords:** faculty teaching, near‐peer teaching, online learning, online teaching, peer‐assisted learning, short answer question

## Abstract

**Introduction:**

Peer‐assisted learning (PAL) is a well‐recognised pedagogical approach in medical education; however, research on its effectiveness in online settings remains limited. Multiple‐choice questions have been the predominant method for assessing PAL outcomes, despite Short Answer Questions (SAQs) being the superior tool for evaluating knowledge. This study compares online peer and faculty teaching in enhancing medical students' higher‐order thinking skills and assesses students' perceptions of these methods.

**Methods:**

Third‐year medical students undergoing surgical placements were consented and recruited for the study. Three pre‐defined cohorts were randomised to the following arms: no intervention (*n* = 41), online PAL teaching (*n* = 37) and online faculty teaching (*n* = 35). Peer teaching was delivered by fourth‐year students (*n* = 6) and faculty teaching by Clinical Teaching Fellows (CTFs) (*n* = 6). Academic outcomes were assessed using end‐of‐block SAQ formatives, and teaching quality was evaluated using the validated SEEQ questionnaire. Knowledge gain and self‐perceived confidence were assessed through pre‐ and post‐session tests, validated with a reference group of learners.

**Results:**

Consent for SAQ exam scores was obtained from *n* = 19 (no intervention), *n* = 29 (PAL) and *n* = 21 (CTF). No significant differences were seen between the groups (*p* = 0.650). SEEQ completion was *n* = 24 (PAL) and *n* = 30 (CTF). CTF tutors received significantly higher ratings in domains of *Learning* (*p* = 0.017) and *Group Interaction* (*p* = 0.036). Pre‐ and post‐session tests showed no significant differences in scores (*p* = 0.957) or self‐perceived confidence ratings (*p* = 0.454).

**Conclusion:**

This study shows that online PAL is a viable alternative to faculty‐led teaching for enhancing SAQ skills and knowledge acquisition. However, faculty‐led teaching offers a superior educational experience.

## 1 Introduction

Short Answer Questions (SAQs) have gained popularity as an assessment tool in medical education, particularly in the form of Very Short Answer Questions (VSAQs) [1]. SAQs require students to construct responses instead of selecting a single correct answer, setting them apart from Multiple‐Choice Questions (MCQs) and Single Best Answer questions (SBAQs) [2]. In medicine, clinical uncertainty is an essential aspect, where a single best answer may not exist. Studies have shown that MCQs may provide a misleading perception of students' understanding by promoting the presence of ‘test‐taking’ and ‘non‐analytical’ behaviours that seem inauthentic to real‐world clinical reasoning [3, 4, 5]. Furthermore, SBAQs and MCQs have been shown to overestimate students' knowledge, making SAQs a more favoured choice for assessing deeper understanding of the subject matter [6, 7, 8, 9, 10]. On the other hand, students have demonstrated a more sophisticated approach when dealing with SAQs, acknowledging uncertainty, employing analytical reasoning and adopting authentic clinical reasoning strategies [11]. Therefore, SAQs offer examiners valuable insight into students' thought processes and enable the assessment of higher‐order thinking skills [12].

MCQs have been shown to overestimate students' knowledge, making SAQs a more favoured choice for assessing deeper understanding.

A promising approach in medical education is ‘peer‐assisted learning’ (PAL), often interchangeably referred to as peer‐teaching, peer‐tutoring or near‐peer teaching [13]. PAL involves more experienced students taking on the role of tutors for their less‐experienced peers; defined as ‘an educational arrangement in which one student teaches one or more fellow students’ [14]. The advantages gained by students from PAL have been understood based on social and cognitive congruence [14, 15, 16]. Social congruence ensures that peer tutors have a greater understanding of the tutees' educational needs and concerns, as they are of similar social backgrounds and ages. Similarly, as the peer tutors and student learners are of similar educational backgrounds and have a shared foundation of knowledge, peer tutors can convey complex and potentially daunting subjects in ways which enhance learners' understanding because of cognitive congruence [16].

Despite the growing prevalence of online learning in the post‐pandemic era and the increasing interest in formally organised PAL [17], the efficacy of PAL in the online environment remains largely understudied. This gap is further highlighted as most research on PAL has concentrated on enhancing medical students' practical skills as opposed to higher‐order thinking skills [18]. Studies that have evaluated students' theoretical knowledge as an outcome of peer teaching have predominantly employed MCQs [13, 19], despite SAQs being the greater tool for assessing students' understanding and deeper comprehension of the subject matter [6, 12]. Furthermore, whilst studies have extensively explored peer teaching versus faculty teaching in traditional settings [13], the specific comparison in the online setting remains limited.

The efficacy of PAL in the online environment remains largely understudied.

### 1.1 Research Aim

In this study, we attempt to address the following questions:

1. Is there a difference in the SAQ performance among students participating in online PAL and online faculty teaching?
2. Is there a difference in the educational quality experienced by students participating in online PAL and online faculty teaching?
3. Do students in online PAL and online faculty groups differ in the acquisition of SAQ skills and their self‐perceived confidence?

## 2 Materials and Methods

This study was approved by The University of Leicester Medicine and Biological Sciences Research Ethics Committee (Ethics Reference: 42308‐gss20‐ls:medicine; PI: Gurtek S Samra).

### 2.1 Participants

Every academic year, the University Hospitals of Leicester NHS Trust hosts surgical placements for third‐year medical students studying at Leicester Medical School, with three blocks scheduled per academic year. Each block has its distinct cohort of students. For this quasi‐experimental study, these pre‐defined student cohorts were randomised to the following arms: block‐one was randomised to no intervention (*n* = 41), block‐two was randomised to online PAL teaching (*n* = 37), and block‐three was randomised to online faculty teaching (*n* = 35).

All third‐year medical students undertaking their surgical rotation at the hospital were invited to participate in this study. Informed consent was obtained from all participants, whilst those not enrolled or who did not consent were excluded from the study.

### 2.2 Tutor Recruitment

The peer tutoring opportunity was advertised to all fourth‐year medical students at the institution, with all applicants (*n* = 6) being selected. Similarly, six on‐site Clinical Teaching Fellows (CTFs) were voluntarily recruited as tutors. Postgraduate experience of the recruited CTFs ranged from 2 to 7 years. Neither the student nor faculty tutors received additional training for the study.

### 2.3 Teaching Programme

An online teaching programme was developed in alignment with the institution's ‘Intended Learning Outcomes’ (ILOs) for the placement. The programme comprised five sessions, each consisting of three interactive, SAQ‐focused, case‐based clinical scenarios (see Table S1). Content and materials were identical for both peer and faculty groups. CTF and PAL tutors were encouraged to supplement the materials with additional explanations and resources at their discretion. During sessions, students collectively engaged with the case‐based SAQ clinical scenarios whilst tutors provided additional explanations and clinical input.

### 2.4 Measured Outcomes

#### 2.4.1 Academic Performance: SAQ Formative Score

To evaluate the academic outcomes of the teaching interventions, anonymised results from the end‐of‐block SAQ formative assessment were analysed. Of the items included in this exam, 19 of 40 marks were dedicated to the content delivered during the online teaching programme.

#### 2.4.2 Educational Quality: SEEQ Score

To assess educational quality, the Student Evaluation of Educational Quality (SEEQ) questionnaire was administered at the end of the teaching interventions. Our modified SEEQ maintained the initial six scales from the original set of nine whilst omitting the final three scales (see Figure S1), as they contained aspects not relevant in this specific context: examinations, assignments and workload [20]. Scores were summed for each domain, serving as an indicator of the educational quality of the teaching interventions [20].

#### 2.4.3 Pre‐/Post‐Session Tests: VSAQs and Confidence

For each teaching session, pre‐ and post‐session questionnaires were administered to students (see Figure S2). Each questionnaire consisted of two sections: the first required students to rate their confidence in answering context‐specific SAQs on a defined 1–5 Likert scale; the second required students to answer VSAQs assessing the ILOs outlined during the sessions.

Akin to the SEEQ scoring, confidence scores were summed for each student to produce a pre‐ and post‐session score, facilitating comparisons within and between intervention groups.

Pre‐ and post‐test VSAQs were developed by the surgical teaching faculty. Post‐test VSAQs differed from pre‐test VSAQs to minimise recollection bias whilst assessing the same ILOs. Pre‐ and post‐tests were found to be of comparable difficulty when utilising a separate group of reference learners (*n* = 34), third‐year medical students who had completed their surgical rotations at separate sites, whose mean ± SD pre‐test score was 18.9 ± 3.9 and mean post‐test score was 19.2 ± 3.9. This difference was not statistically significant with a mean difference of 0.2 ± 3.3 (95% CI: −0.95 to 1.39; *p* = 0.70), confirming that the pre‐ and post‐tests were of similar difficulty. Furthermore, internal consistency reliability was evaluated using Cronbach's alpha. The pre‐test had a Cronbach's alpha value of 0.70, and the post‐test Cronbach's alpha was 0.75, indicating acceptable reliability [21]. The combined Cronbach's alpha for all pre‐ and post‐test items was 0.83, suggesting good overall internal consistency [21].

### 2.5 Statistical Methods

SAQ scores were analysed with ANOVA, SEEQ domains with *t*‐tests or Mann–Whitney *U* tests, and pre‐/post‐session changes with paired *t*‐tests; group differences used independent *t*‐tests (see supplemental material).

## 3 Results

### 3.1 SAQ Formative Scores

Nineteen of the 41 students (46.3%) in the no‐intervention group, 29 of 37 student tutees (78.4%) in the PAL group, and 21 of 35 student tutees (60%) in the CTF group consented to share their anonymised SAQ scores for this study. Though the PAL group exhibited the highest mean score, the differences between the groups were not statistically significant (*p* = 0.650) (Table 1).

**TABLE 1:** Comparison of formative SAQ scores between no‐intervention, PAL and CTF groups, expressed as mean ± standard deviation.

	No‐intervention group, mean ± SD (*n* = 19)	PAL group, mean ± SD (*n* = 29)	CTF group, mean ± SD (*n* = 21)	*p*
SAQ score (max 40 marks)	25.6 ± 2.8	26.1 ± 2.9	25.4 ± 3.2	0.650

Abbreviations: CTF = clinical teaching fellow; PAL = peer‐assisted learning; SAQ = short answer question; SD = standard deviation.

### 3.2 SEEQ

Twenty‐four of the 37 students (64.8%) in the PAL group and 30 of the 35 students (85.7%) in the CTF group completed the modified SEEQ at the end of their respective teaching programmes.

In the *Learning* domain, the CTF group scored a mean score of 18 ± 1.9 versus 16.8 ± 1.8 in the PAL group; this difference was statistically significant (*p* = 0.017). Of the items in this domain, the CTF group scored significantly higher on SEEQ statements three and four: *My interest in the subject has increased as a consequence of this course* (*p* = 0.048); *I have learned and understood the subject materials of this course* (*p* = 0.049).

In the *Group Interaction* domain, the median [IQR] score of the CTF group was 19 [16, 17, 18, 19, 20], compared with 16 [16, 17, 18, 19, 20] for the PAL group; this difference was statistically significant (*p* = 0.036). Of the items in this domain, statements 14 and 15 had significantly higher scores compared to the PAL group: *Students were invited to share their ideas and knowledge* (*p* = 0.028); *Students were encouraged to ask questions and were given meaningful answers* (*p* = 0.018).

No significant differences were observed in the remaining domains, including Organisation (*p* = 0.543), Breadth (*p* = 0.459), Enthusiasm (*p* = 0.765), Individual Rapport (*p* = 0.234), and the Overall total score (*p* = 0.194) (Table 2).

**TABLE 2:** Comparison of SEEQ scores between PAL and CTF groups.

SEEQ domain	PAL (*n* = 24)		CTF group (*n* = 30)		
	Median [IQR]	Mean ± SD	Median [IQR]	Mean ± SD	*p*
Learning	—	16.8 ± 1.8	—	18 ± 1.9	0.017*
Enthusiasm	—	17.2 ± 2.4	—	17 ± 2.5	0.765
Organisation	18.5 [16–20]	—	19 [16–20]	—	0.543
Group interaction	16 [16–20]	—	19 [16–20]	—	0.036*
Individual rapport	16.5 [15–20]	—	18.5 [16–20]	—	0.234
Breadth	16 [15–20]	—	16 [16–20]	—	0.459
Overall total	—	102.5 ± 11.3	—	106.3 ± 11	0.194

*Note:* Data are presented as mean ± standard deviation or median [interquartile range], as appropriate.

Abbreviations: CTF = clinical teaching fellow; IQR = interquartile range; PAL = peer‐assisted learning; SEEQ = Student Evaluation of Educational Quality; SD = standard deviation.

* Significant at *p* < 0.05.

### 3.3 Pre‐ and Post‐Session Tests

In the analysis of VSAQ scores, both the PAL and CTF groups showed a significant improvement in scores for sessions one and five (*p* < 0.05). Only the PAL group showed a significant improvement in scores for session two (*p* < 0.05). No significant differences in scores were observed for sessions three and four in either group (see Table S3).

For the self‐perceived confidence in answering surgical SAQs, for session two, the CTF group did not exhibit a significant difference in confidence (*p* = 0.137). For all other sessions, both PAL and CTF groups demonstrated significant improvements in confidence (*p* < 0.05) (see Table S4).

The mean difference in VSAQ pre‐ and post‐session test scores between the PAL and CTF groups was not significant (*p* = 0.957), suggesting that both groups achieved similar learning outcomes as measured by the VSAQs. Additionally, the mean difference in self‐perceived confidence was also insignificant (*p* = 0.454), implying that both PAL and CTF tutors were equally effective in enhancing students' confidence in their surgical knowledge (Table 3).

**TABLE 3:** Independent sample *t*‐test for mean comparison of % difference between PAL and CTF groups in pre‐ and post‐session tests, expressed as mean ± standard deviation.

Metric	PAL group, mean ± SD	CTF group, mean ± SD	*p*
VSAQ score	26 ± 16.3	25.5 ± 12.2	0.957
Confidence score	19.5 ± 3.7	17.3 ± 4.7	0.454

Abbreviations: CTF = clinical teaching fellow; PAL = peer‐assisted learning; SD = standard deviation; VSAQ = very short answer question.

## 4 Discussion

In this study, we assessed the effects of online peer teaching and online faculty teaching on students' SAQ skills and perceived confidence, whilst also assessing the educational quality of these interventions. Previous meta‐analyses have shown PAL to be as equally effective as traditional faculty teaching when assessing clinical skills and knowledge outcomes, often exhibiting no significant differences [13, 18, 19]. However, it is noteworthy that many of the studies within these analyses have measured clinical knowledge outcomes using MCQs as opposed to SAQs. Furthermore, these teaching approaches have been extensively explored using traditional in‐person settings, whilst research in the online environment remains limited despite the growing reliance on online teaching methods. This study uniquely contributes to the field and addresses this gap by evaluating online PAL and online faculty‐led teaching, using SAQs—an under‐utilised yet robust tool for assessing higher‐order thinking and comprehension. Furthermore, to provide additional context to our primary outcome measures, we also assessed educational quality using the validated SEEQ questionnaire.

In this study, we found SAQ outcomes to be comparable for both faculty and peer teaching groups. A previous study conducted by Kassab et al. [22] compared problem‐based learning (PBL) tutorials led by faculty to peer tutors and found no significant differences in the end‐of‐unit SAQ summative exams. The authors acknowledged that in the context of PBL learning, the use of SAQ assessments as an outcome measure may have been a limitation itself, as it is a measure of ‘knowledge construct, whilst the tutorial process emphasises other aspects of the PBL process’. In our study, we assessed the effects of an online SAQ‐focused teaching programme delivered by either peer or faculty tutors and found no significant differences in both the end‐of‐block assessments and pre‐ and post‐session test scores.

We found SAQ outcomes to be comparable for both faculty and peer teaching groups.

An interesting finding in our study was that there was no significant difference in SAQ scores between the no‐intervention and teaching groups. Batchelder et al. [23] compared the summative results for students attending a peer‐led programme (*n* = 310) to those who did not (*n* = 48). No significant differences were seen between the two groups for SAQ and SBA scores. However, it is important to note that both our study and that by Batchelder et al. have notable limitations, such as the lack of randomisation at the individual level and disparity in participant numbers between the study arms. The disparity in sample sizes between groups, with fewer participants in the no‐intervention group, may have reduced the statistical power of our analysis, potentially masking the effects of the teaching interventions. Additionally, individual student factors such as prior knowledge, study habits, and motivation could have influenced the outcomes, making it more challenging to detect differences between groups. To address these challenges, future educational research should employ individual randomisation and matching to ensure greater comparability between groups. Furthermore, analysing qualitative data on student engagement, motivation and study habits could provide valuable insights into how these factors shape learning outcomes, helping contextualise and explain the findings more comprehensively.

The SEEQ questionnaire has shown its reliability, efficacy and validity as a tool for measuring the quality of education in many studies [24, 25, 26, 27, 28]. Furthermore, the questionnaire's subdomains have also demonstrated high internal consistency [29]. Utilising the tool, we found CTF tutors to receive significantly higher ratings in the *Learning* and *Group Interaction* domains, thus suggesting a higher‐quality educational experience for the faculty group students, a finding consistent with the literature [30, 31, 32]. In a randomised controlled trial aiming to teach students ultrasound skills, faculty tutors were rated higher than peer tutors on items relating to fun and competency [32]. Furthermore, Heckmann et al. [30] assessed the effects of PAL for clinical skills training, with peer tutors' competence being rated lower than faculty tutors. These findings can be attributable to the greater experience, knowledge and refined teaching skills of faculty tutors. Additionally, the professional experience and expertise may inspire greater confidence and respect from students, contributing to higher perceived educational quality.

We found CTF tutors to receive significantly higher ratings in the *Learning* and *Group Interaction* domains.

Although there were no significant differences in academic outcomes between groups, the responses on the SEEQ indicate a greater benefit to CTF teaching. We believe these findings reflect differences in the ‘hidden curriculum’, the implicit facets of learning and teaching which involve the acquisition of perceptions and lessons not explicitly covered in the formal curriculum [33]. Compared with the PAL group, students in the CTF group felt more *invited to share their ideas and knowledge* (SEEQ statement 14) and more *encouraged to ask questions and were given meaningful answers* (SEEQ statement 15). With the CTF group reporting a higher quality educational experience, it appears that the particular teaching style and approach of faculty tutors may have fostered a deeper understanding of the subject matter, as evidenced by faculty tutees scoring higher on statement four: *I have learned and understood the subject materials of this course*. Furthermore, these positive interactions with role models could have further‐reaching effects on learners, such as influencing career decisions [34], as shown by the significantly higher ratings on statement three: *My interest in the subject has increased as a consequence of this course*. However, it is worth considering that students in the CTF group would have interacted with tutors outside of the online teaching programme, such as during group work and bedside teaching sessions throughout the block. Whilst the SEEQ was specifically used to assess the online teaching programme, these existing relationships could have influenced SEEQ responses.

Although there were no significant differences in academic outcomes … responses on the SEEQ indicate a greater benefit to CTF teaching.

### 4.1 Strengths and Limitations

The strengths of our study lie in the comprehensive assessment methods used to evaluate the outcomes of the teaching interventions. For our primary outcome measure, we opted to use formative assessments instead of summative assessments. Raupach et al. [35] revealed that using summative exams to assess academic outcomes could *mask* the influence of PAL interventions. The study compared near‐peer teaching to traditional lectures, assessing student outcomes in two cohorts. A summative exam was used to assess the student outcomes in the first cohort, whereas a formative exam was used for the second cohort. Only in the second cohort was a significant advantage of peer‐teaching over lectures observed. On the other hand, for the cohort in which the summative assessment was utilised, there were no significant differences between PAL and traditional lecture teaching. The authors concluded that ‘assessment format appeared to be more powerful than the choice of instructional method in enhancing student learning’, with the summative exam creating ‘a massive additional learning incentive, regardless of randomisation’. Furthermore, to assess knowledge gain, we employed pre‐ and post‐session tests, which were shown to be of comparable difficulty and good internal consistency. Moreover, we used a validated questionnaire to measure and compare the quality of education for the intervention groups.

Despite the strengths, our study has several limitations. Firstly, an individually randomised double‐blinded study design was not feasible due to logistic and organisational constraints. Therefore, we opted for group randomisation as the most suitable alternative, classifying this study as quasi‐experimental. Additionally, there was a disparity in the number of participants consenting to share their anonymised SAQ formative results between the study arms. This disparity might have influenced the outcomes and reduced our ability to detect subtle differences between groups. Moreover, there was a discrepancy between the number of students attending the sessions and filling both pre‐ and post‐session tests (see Table S2). Furthermore, the study was conducted at a single institution with a specific cohort of third‐year medical students undertaking a surgical rotation, potentially limiting the generalisability of the findings to other institutions, student populations or clinical settings. Although qualitative data in the form of post‐session comments and feedback were gathered, future work entailing comprehensive qualitative data acquisition and analysis will provide further insight into the benefits and differences between online PAL and CTF teaching.

## 5 Conclusion

This study shows that online PAL can be an effective alternative to online faculty‐led teaching, yielding similar academic outcomes in SAQ performance and self‐perceived confidence. However, faculty tutors may provide a higher‐quality educational experience for students, therefore, where resources are limited, online PAL offers a viable option for enhancing student learning whilst maintaining comparable academic results.

## Author Contributions

**Gurtek Singh Samra:** writing – original draft, writing – review and editing, software, formal analysis, project administration, data curation, investigation, resources, conceptualization, methodology, validation, visualization. **Kashmir Gaddu:** writing – review and editing, resources, conceptualization, visualization, formal analysis, validation, methodology, investigation. **Joseph Ryan Wong Sik Hee:** project administration, writing – review and editing, visualization, methodology, validation, investigation. **Krupali Brahmbhatt:** writing – review and editing, methodology, visualization, project administration, validation. **David Bowrey:** conceptualization, methodology, writing – review and editing, validation, visualization. **Max Seabrook:** supervision, writing – review and editing, methodology, conceptualization, validation, visualization.

## Ethics Statement

This study was approved by the University of Leicester's Medicine and Biological Sciences Research Ethics Committee (Ethics Reference: 42308‐gss20‐Is: medicine; Research Project Title: Near‐Peer Teaching versus Clinical Teaching Fellows in Surgical SAQ Teaching for 3rd‐year medical students on their surgical rotation; PI: Gurtek S Samra).

## Conflicts of Interest

The authors declare no conflicts of interest.

## Supporting information


**Figure S1.** SEEQ used in our study.
**Figure S2.** An exemplar confidence question used in the pre‐ and post‐forms for session two of the teaching programme (a); 2 of 6 exemplar VSAQs used in the pre‐session form for session two of the teaching programme (b).
**Table S1.** Clinical SAQ cases covered during the online programme, with corresponding dates for PAL and Faculty‐led sessions.
**Table S2.** Attendance for the online teaching sessions, as per MS Teams generated Attendence reports.
**Table S3.** Paired t‐test for comparison of VSAQ scores (%) between PAL and CTF groups, expressed as mean ± standard deviation.
**Table S4.** Paired t‐test for comparison of self‐perceived confidence scores (%) between PAL and CTF groups, expressed as mean ± standard deviation.

## Data Availability

The data that support the findings of this study are available on request from the corresponding author. The data are not publicly available due to privacy or ethical restrictions.
