# Supplementary material for: Comparison of Online Peer‐Assisted Learning and Faculty‐Led Teaching for Short Answer Questions
Source: Clin Teach. 2025 Mar 18;22(3):e70069. doi: 10.1111/tct.70069 (PMC11920381; doi:10.1111/tct.70069)
Supplement: Supplementary file 1 — Figure S1. SEEQ used in our study. Figure S2. An exemplar confidence question used in the pre‐ and post‐forms for session two of the teaching programme (a); 2 of 6 exemplar VSAQs used in the pre‐session form for session two of the teaching programme (b). Table S1. Clinical SAQ cases covered during the online programme, with corresponding dates for PAL and Faculty‐led sessions. Table S2. Attendance for the online teaching sessions, as per MS Teams generated Attendence reports. Table S3. Paired t‐test for comparison of VSAQ scores (%) between PAL and CTF groups, expressed as mean ± standard deviation. Table S4. Paired t‐test for comparison of self‐perceived confidence scores (%) between PAL and CTF groups, expressed as mean ± standard deviation. [file TCT-22-e70069-s001.docx]

# Supplemental Material

Contents

[Statistical Methods 1](#_Toc184809380)

[SAQ Formative scores 1](#_Toc184809381)

[SEEQ scores 1](#_Toc184809382)

[Pre- and post-session tests 1](#_Toc184809383)

[Figures 2](#_Toc184809384)

[Tables 4](#_Toc184809385)

## List of Figures

[Figure 1. SEEQ used in our study. 2](#_Toc184810658)

[Figure 2. An exemplar confidence question used in the pre- and post-forms for session two of the teaching programme (a); 2 of 6 exemplar VSAQs used in the pre-session form for session two of the teaching programme (b). 3](#_Toc184810659)

## List of Tables

[Table 1. Clinical SAQ cases covered during the online programme, with corresponding dates for PAL and Faculty-led sessions. 4](#_Toc184810653)

[Table 2. Attendance for the online teaching sessions, as per MS Teams generated Attendence reports. 4](#_Toc184810654)

[Table 3. Paired t-test for comparison of VSAQ scores (%) between PAL and CTF groups, expressed as mean ± standard deviation. 4](#_Toc184810655)

[Table 4. Paired t-test for comparison of self-perceived confidence scores (%) between PAL and CTF groups, expressed as mean ± standard deviation. 5](#_Toc184810656)

# Statistical Methods

## SAQ Formative scores

A one-way ANOVA was conducted for SAQ scores across the no-intervention, PAL,

and CTF groups. The Shapiro-Wilk test was used to confirm the normality of the data,

and the assumption of equal variances was also met.

## SEEQ scores

An independent samples t-test was conducted for domains that demonstrated a normal distribution, as determined by the Shapiro-Wilk test of normality. The Mann-Whitney U test was used to compare groups for domains where normality was not observed.

## Pre- and post-session tests

Meeting the necessary assumptions, paired t-tests were conducted to assess for changes

in VSAQ scores and confidence scores within each teaching session for both CTF and

PAL groups. The mean differences for VSAQ and confidence scores were compared

using an independent sample t-test to determine if there were any significant differences

between the PAL and CTF groups.

A p-value of less than 0.05 was considered statistically significant for all tests.

All statistical analysis was conducted using Stata 18 (StataCorp. 2023. Stata Statistical

Software: Release 18. College Station, TX: StataCorp LLC.).

# Figures


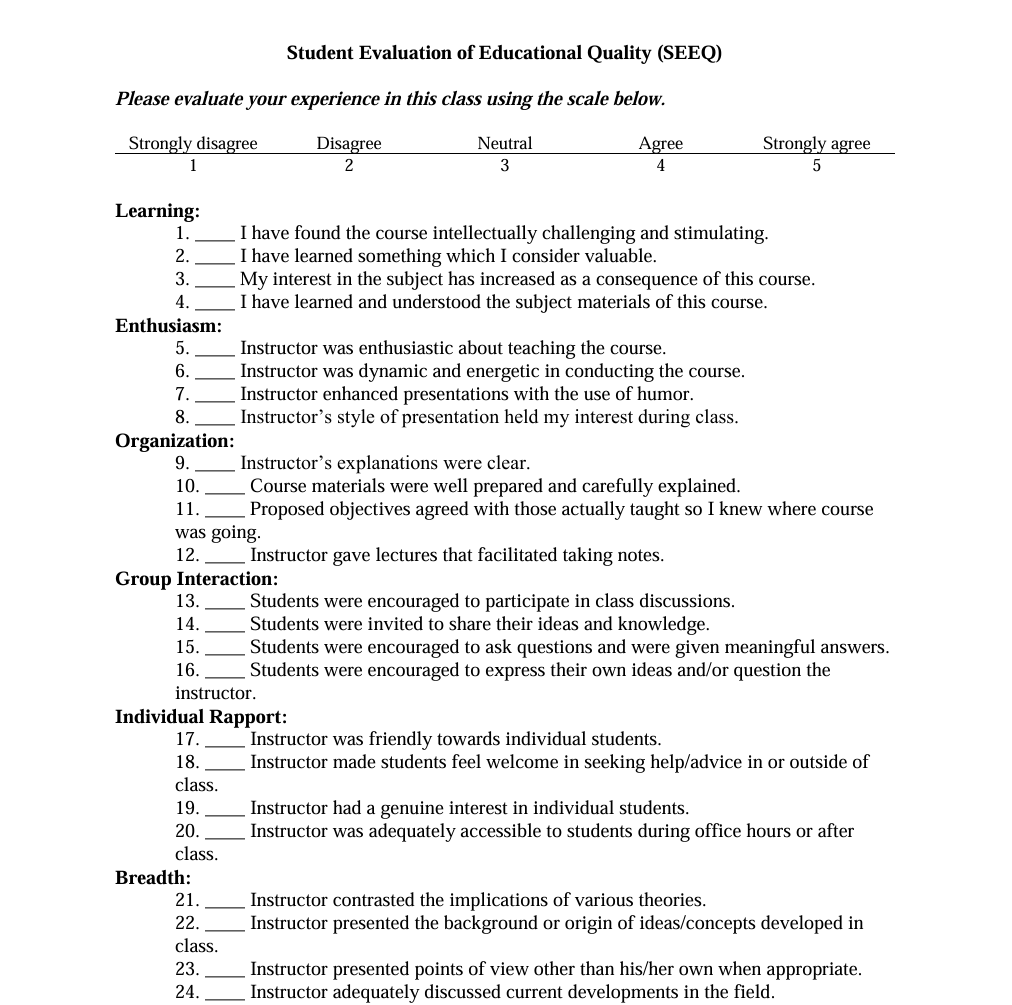


Figure 1. SEEQ used in our study.


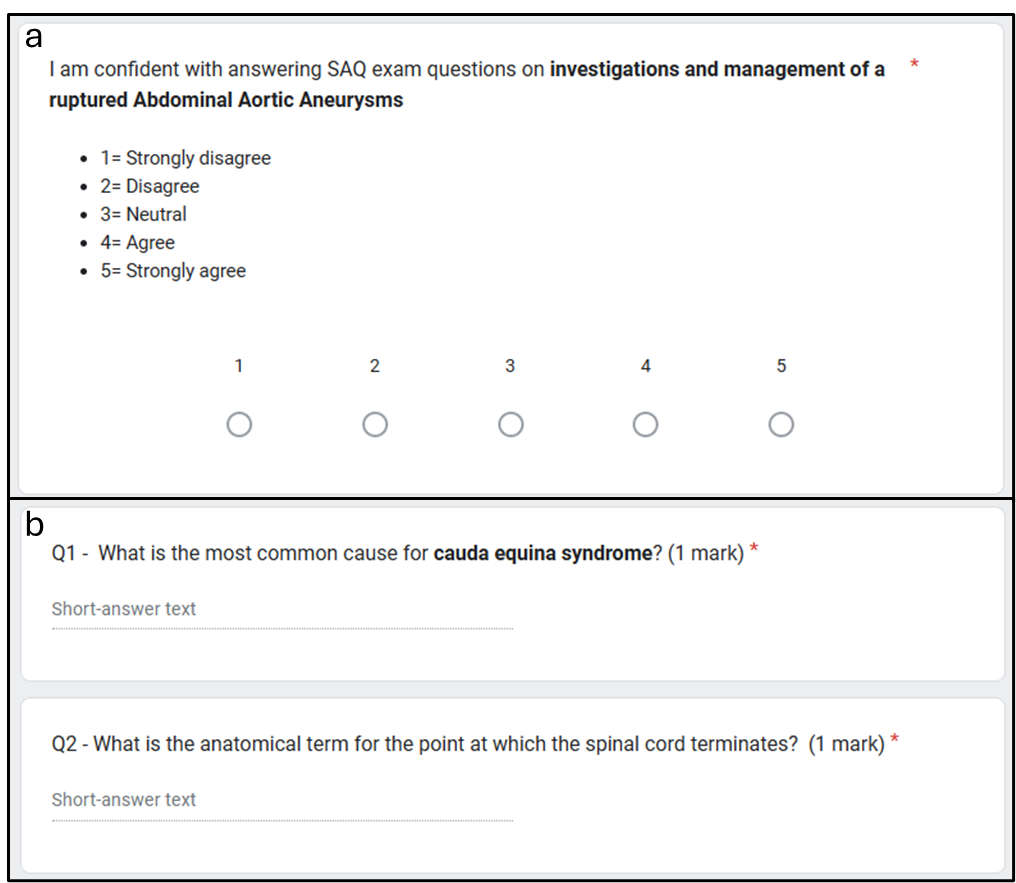


Figure 2. An exemplar confidence question used in the pre- and post-forms for session two of the teaching programme (a); 2 of 6 exemplar VSAQs used in the pre-session form for session two of the teaching programme (b).

# Tables

Table 1. Clinical SAQ cases covered during the online programme, with corresponding dates for PAL and Faculty-led sessions.

| Session | Topics | | | PAL | CTF |
| --- | --- | --- | --- | --- | --- |
| 1 | General surgery:  Bowel Cancer | T&O:  Hip fractures | Urology:  Torsion | 23/01/24 | 16/04/24 |
| 2 | Vascular:  AAA | T&O:  Cauda equina | General surgery:  Bowel obstruction | 30/01/24 | 23/04/24 |
| 3 | T&O:  Ankle fractures | Urology:  Renal colic | General surgery:  Cholangitis | 06/02/24 | 30/04/24 |
| 4 | T&O:  Open fractures | General surgery:  Gastric cancer | Peri-op:  Blood Transfusion | 13/02/24 | 07/05/24 |
| 5 | Breast: Cancer | Vascular:  PAD | Peri-op:  STOP/START meds | 20/02/24 | 14/05/24 |

Table 2. Attendance for the online teaching sessions, as per MS Teams generated Attendence reports.

|  | Attendees | |
| --- | --- | --- |
| Session number | PAL group | CTF group |
| Session 1 | 26 | 31 |
| Session 2 | 19 | 32 |
| Session 3 | 25 | 24 |
| Session 4 | 13 | 23 |
| Session 5 | 18 | 18 |

Table 3. Paired t-test for comparison of VSAQ scores (%) between PAL and CTF groups, expressed as mean ± standard deviation.

| Teaching Session | Group | Pre-test %, mean ± SD | Post-test %, mean ± SD | Difference, mean ± SD | p-value |
| --- | --- | --- | --- | --- | --- |
| 1 | PAL (n = 11) | 60.6 ± 22.7 | 90.9 ± 22.5 | 30.3 ± 26.7 | 0.004* |
|  | CTF (n = 11) | 40.2 ± 31.4 | 83.3 ± 16.7 | 43.2 ± 36.7 | 0.003* |
| 2 | PAL (n = 8) | 40.6 ± 22.3 | 91.7 ± 13.4 | 51 ± 32.3 | 0.003* |
|  | CTF (n = 5) | 51.7 ± 33 | 78.3 ± 31 | 26.7 ± 52.5 | 0.320 |
| 3 | PAL (n = 19) | 68.4 ± 16.6 | 75.4 ± 18.7 | 7 ± 22.4 | 0.190 |
|  | CTF (n = 7) | 65.5 ± 14 | 76.2 ± 28.6 | 10.7 ± 21.9 | 0.243 |
| 4 | PAL (n = 9) | 49.1 ± 29 | 68.5 ± 26.9 | 19.4 ± 38.2 | 0.165 |
|  | CTF (n = 5) | 48.3 ± 19 | 66.7 ± 26.4 | 18.3 ± 25.3 | 0.180 |
| 5 | PAL (n = 9) | 50.9 ± 16.4 | 73.2 ± 22 | 22.2 ± 13.8 | 0.001* |
|  | CTF (n = 7) | 45.2 ± 13.5 | 73.8 ± 13.1 | 28.6 ± 11.6 | <0.001* |

*Significant at p < 0.05; PAL = peer-assisted learning; CTF = clinical teaching fellow; SD = standard deviation

Table 4. Paired t-test for comparison of self-perceived confidence scores (%) between PAL and CTF groups, expressed as mean ± standard deviation.

| Teaching Session | Group | Pre-test %, mean ± SD | Post-test %, mean ± SD | Difference, mean ± SD | p-value |
| --- | --- | --- | --- | --- | --- |
| 1 | PAL (n = 11) | 61.4 ± 12.3 | 83.6 ± 8.1 | 22.3 ± 10.1 | <0.001* |
|  | CTF (n = 11) | 50.5 ± 10.1 | 73.6 ± 14 | 23.2 ± 9.6 | <0.001* |
| 2 | PAL (n = 8) | 70.5 ± 7.4 | 85 ± 6 | 14.5 ± 7.1 | <0.001* |
|  | CTF (n = 5) | 66.4 ± 11.5 | 76.8 ± 9.6 | 10.4 ± 12.5 | 0.137 |
| 3 | PAL (n = 19) | 62.9 ± 9.6 | 79.5 ± 6.9 | 16.6 ± 10.2 | <0.001* |
|  | CTF (n = 7) | 59.3 ± 17.4 | 75 ± 19.4 | 15.7 ± 13.4 | 0.021* |
| 4 | PAL (n = 9) | 56.1 ± 10.8 | 77.8 ± 9.4 | 21.7 ± 11.5 | <0.001* |
|  | CTF (n = 5) | 59 ± 10.8 | 77 ± 14.4 | 18 ± 10.4 | 0.018* |
| 5 | PAL (n = 9) | 56.9 ± 13.8 | 79.1 ± 12.8 | 22.2 ± 12.2 | <0.001* |
|  | CTF (n = 7) | 56.6 ± 16.6 | 76 ± 14.1 | 19.4 ± 6.3 | <0.001* |

*Significant at p < 0.05; PAL = peer-assisted learning; CTF = clinical teaching fellow; SD = standard deviation
